# Perspective Shape-from-Shading Problem: A Unified Convergence Result for Several Non-Lambertian Models

**DOI:** 10.3390/jimaging8020036

**Published:** 2022-02-01

**Authors:** Silvia Tozza

**Affiliations:** Department of Mathematics, Alma Mater Studiorum—Università di Bologna, Piazza di Porta San Donato 5, 40126 Bologna, Italy; silvia.tozza@unibo.it

**Keywords:** Shape-from-Shading, perspective projection, convergence result, non-Lambertian models, stationary Hamilton–Jacobi equations

## Abstract

Shape-from-Shading represents the problem of computing the three-dimensional shape of a surface given a single gray-value image of it as input. In a recent paper, we showed that the introduction of an attenuation factor in the brightness equations related to various perspective Shape-from-Shading models allows us to ensure the well-posedness of the corresponding differential problems. Here, we propose a unified convergence result of a numerical scheme for several non-Lambertian reflectance models. This result is interesting since it can be easily extended to other non-Lambertian models in a unified and, therefore, powerful framework.

## 1. Introduction

The Shape-from-Shading (SfS) problem consists of computing the three-dimensional shape of an object starting from a single gray-level image of it. This problem aroused the interest of some opticians in the 1950s–1960s [1,2], and then the problem was formulated by B.K.P. Horn and his collaborators of MIT as a first-order non-linear partial differential equation (PDE) in the 1970s–1980s [3,4,5]. The goal was to enable the 3D surface represented in the input image to solve a Hamilton–Jacobi (HJ) equation or a variational problem. This problem gave rise to an expansion in the field of mathematics, and some researchers tried to prove the well-posedness of it in the framework of weak solutions. The first works of Lions, Rouy and Tourin in the early 1990s [6,7] inserted the SfS problem into the context of the viscosity solutions frameworks, hence in a much more theoretical area.

In general, the SfS problem is described by the following image irradiance equation [8]:

(1) I(x)=R(N(x)).

In this equation, the gray-level intensity I(x) of the given input image at the point x:=(x,y) is put in relation to the reflectance function R(N(x)), which represents the value of the light reflection on the surface as a function of its orientation, i.e., of the unit normal N(x). Different expressions of the function *R* produce different reflectance models.

There are three main ingredients for formulating the SfS problem: how we model the light, the camera and the reflectance. The most common setup found in the literature considers the Lambertian reflectance model (for which the intensity I(x) depends only on the incident angle between the direction of the light source ω and the direction of the normal unit vector, without considering the viewer), under an orthographic projection (no perspective deformations are considered) with a single light source located far from the object to be reconstructed (located *at infinity* in the direction of the light versor ω). In this case, the irradiance Equation (1) can be written as the following HJ equation:

(2) $$I\left(x\right)=\gamma_{D}\frac{\omega \cdot (-\nabla u(x),1)}{\sqrt{1+{\left|\nabla u\left(x\right)\right|}^{2}}},$$

where *u* is the unknown surface height and $\gamma_{D}$ is the diffuse albedo, which indicates the reflective power of a diffuse surface.

In general, non-linear PDEs such as (2) do not admit smooth solutions. Hence, we need to look for a proper notion of a weak solution, for example, in the context of the viscosity ones (see [9,10,11]). Unfortunately, under orthographic projection, there is no uniqueness of solution, not only in the classical sense but also in the weak sense, due to the well-known concave/convex ambiguity related to singular points, i.e., points where the intensity of the image is at the maximum and the light direction is parallel to the surface normal, generating countless viscosity solutions to Equation (2). In order to overcome this ambiguity, several attempts have been made by the scientific community in different research directions. Considering a priori information on the object shape model and/or an integrability condition of the height field in order to constrain the ambiguities could be a possibility. From the mathematical point of view, adding information considering more than one input image, taken from the same point of view but with different light sources (photometric-stereo technique [12,13]) or under the same light source but with different viewpoints (stereovision; see [14]), allows us to achieve well-posedness. In order to stay within the scope of a single input image, a perspective Lambertian model was proposed in [15,16,17], with a light source located far from the surface to reconstruct, i.e., located at infinity. In [16], and then in the extended journal version [17] by the same authors, it was observed that the perspective projection is a more realistic projection than the orthographic one, and it improves the performance with respect to considering the orthographic projection, regardless of the specific numerical algorithm employed (in [17], a comparison with [15] and three orthographic SfS methods is also reported, in order to show the better performance of the perspective models). In [18,19], a different setup for the perspective model was proposed, with a pinhole camera and light source located at the optical center. All these works are limited to the assumption of a Lambertian reflectance model, which is known to be not always suitable for describing real-world surfaces. Models for non-Lambertian surfaces (e.g., [20,21,22]) have been proposed in chronologically later works [23,24,25,26]. All these models, when formulated in terms of a differential equation, do not resolve the concave/convex ambiguity (see [27] for an analysis of them). In order to achieve the uniqueness of solution starting from a single input image, in [18,28], the authors introduced an attenuation factor in the Lambertian brightness equation under perspective projection. This factor takes into account the distance between the surface and the light source and, thanks to this attenuation term, the associated HJ equation admits a unique viscosity solution. Starting from the idea in [18] related to the Lambertian model, in [29], the authors showed the well-posedness of several non-Lambertian models in a unified formulation, by validating the assumptions of the *Maximum Principle for discontinuous viscosity solutions* (see [9]). Many works have focused on the approximation of the different brightness equations derived from the various models considered for solving the SfS problem (the reader can refer to the surveys [30,31] for more details), but only very few of them treat the convergence of the corresponding methods, and the results are often of experimental type. In this paper, we show the convergence of a numerical scheme for the perspective SfS problem associated with different non-Lambertian models, based on the method proposed in [32] and the theoretical results contained in [29]. To the best of our knowledge, this is the first paper in which a theoretical convergent result of a numerical scheme related to different non-Lambertian models in a unified framework is provided.

The paper is organized as follows: in Section 2, we introduce the setup and the notation adopted, briefly recalling the reflectance models we considered and how it is possible to write them in a unified formulation. Then, we recall the definition and basic properties of viscosity solutions and the theoretical results shown in [29]. Section 3 represents the core of this work, in which we state and prove the convergence result. In Section 4, numerical experiments confirm the effectiveness of the proposed scheme. The paper ends with final comments and conclusions reported in Section 5.

## 2. Preliminary Materials and Models

In this section, we introduce the notation and the setup we adopted, before recalling the four reflectance models considered. Finally, we introduce the definition of viscosity solutions and their basic properties.

### 2.1. Setup and Notation

For all four models, for the setup, we used the perspective camera projection with one light source located at the optical center of the camera and an attenuation term of the illumination due to the distance between the surface and the light source.

Let $\Omega \subset R^{2}$ be open and bounded, which represents the image domain. As performed in [18,23,25,28], the scene can be represented by a surface $S:\bar{\Omega }\to R^{3}$ defined as the following function:

(3) $$S\left(x\right)=\frac{fu(x)}{\sqrt{{\left|x\right|}^{2}+f^{2}}}\left(x,-f\right)$$

where u(x) is the unknown height of the surface and f>0 is the focal length of the camera. The unit vector in the direction of the light source is defined as follows:

(4) $$\omega \left(S\left(x\right)\right)=\frac{\left(-x,f\right)}{\sqrt{{\left|x\right|}^{2}+f^{2}}}\;.$$

The unit normal vector at the point x of S is defined as $N\left(x\right)=\frac{n(x)}{\left|n(x)\right|}$, where

(5) $$\;n\left(x\right)=S_{x}\times S_{y}=(f\;\nabla u\left(x\right)-\frac{fu(x)}{{\left|x\right|}^{2}+f^{2}}\;x,\nabla u\left(x\right)\cdot x+\frac{fu(x)}{{\left|x\right|}^{2}+f^{2}}\;f)$$

and

(6) $$\begin{array}{cc} |n(x)|= & \sqrt{f^{2}{\left|\nabla u\left(x\right)\right|}^{2}+{\left(\nabla u\left(x\right)\cdot x\right)}^{2}+u{\left(x\right)}^{2}\frac{f^{2}}{\left(f^{2}+|x{|}^{2}\right)}}. \end{array}$$

### 2.2. Reflectance Models

We now briefly recall the reflectance models and their corresponding HJ equations, describing them in a unified formulation (see [29] for more details).

#### 2.2.1. Lambertian Model

The Lambertian model (L-model) is the most common and simple reflectance model ([4,5,18,19]), which does not consider the viewer position. The brightness equation for this model, considering an attenuation term, is as follows:

(7) $$I\left(x\right)=\gamma_{D}\left(x\right)\frac{\cos(\theta_{i})}{r^{2}}$$

where I(x) denotes the intensity of the image, $\gamma_{D}\left(x\right)$ is the diffuse albedo, $\theta_{i}$ is the incident angle, i.e., the angle between the light source direction ω(S(x)) and the unit normal to the surface N(x), and $1/r^{2}$ is the attenuation term of the illumination due to the distance between the surface and the light source [23]. Under the setup introduced in Section 2.1, supposing $\gamma_{D}\left(x\right)$ = 1, i.e., all incident light is reflected, and considering the attenuation term $1/r^{2}$, with r=fu(x), the following HJ equation can be associated with (7):

(8) $$I\left(x\right)=\frac{1}{f^{2}u{\left(x\right)}^{2}}\frac{u(x)Q(x)}{\sqrt{f^{2}{\left|\nabla u\left(x\right)\right|}^{2}+{\left(\nabla u\left(x\right)\cdot x\right)}^{2}+u{\left(x\right)}^{2}Q{\left(x\right)}^{2}}}$$

where

(9) $$Q\left(x\right):=\frac{f}{\sqrt{f^{2}+{\left|x\right|}^{2}}}.$$

By defining

(10) $$W\left(x,p\right):=\frac{f^{2}{\left|p\right|}^{2}+{\left(p\cdot x\right)}^{2}}{Q^{2}},$$

we can rewrite Equation (8) as follows:

$$I\left(x\right)=\frac{1}{f^{2}u{\left(x\right)}^{2}}\frac{u(x)}{\sqrt{W\left(x,\nabla u\left(x\right)\right)+u{\left(x\right)}^{2}}}.$$

Supposing that the surface S is in front of the optical center, i.e., it is visible, then *u* is a strictly positive function. Hence, we can consider the change of variable v(x)=lnu(x), arriving at the following HJ equation in the new variable *v* (cf. [18,28])

(11) $$-e^{-2v(x)}+I\left(x\right)f^{2}\sqrt{W(x,\nabla v(x))+1}=0,$$

to which we associate the Hamiltonian

(12) $$H^{L}\left(x,s,p\right)=-e^{-2s}+I\left(x\right)f^{2}\sqrt{W(x,p)+1}\;.$$

#### 2.2.2. Oren–Nayar Model

Considering real-world surfaces, e.g., plaster, sand, or concrete, the Lambertian model is an inadequate approximation of the diffuse component, mainly because it does not take into account the roughness of the surface. In the 1990s, Michael Oren and Shree K. Nayar proposed a different diffuse model, suitable for rough surfaces, which are modelled as a set of facets with different slopes, each of them Lambertian in reflectance [22,33]. This model takes into account complex physical phenomena, such as masking, shadowing and interreflections between points on the surface facets, and can be viewed as a generalization of the Lambertian one. The brightness equation for the Oren–Nayar model (ON-model) is as follows:

(13) $$I\left(x\right)=\cos\left(\theta_{i}\right)\left(A+B\sin\left(\alpha \right)\tan\left(\beta \right)\max\left\{0,\cos\left(\varphi_{r}-\varphi_{i}\right)\right\}\right),$$

where $\theta_{i}$ indicates the incident angle, $\theta_{r}$ denotes the angle between the observer V and the normal N directions, α and β are defined as $\alpha =\max\left\{\theta_{i},\theta_{r}\right\}\;\mathrm{and}\;\beta =\min\left\{\theta_{i},\theta_{r}\right\}$, respectively. $\varphi_{i}$ and $\varphi_{r}$ are, respectively, the angles between the projection of the light source direction ω, or the projection of the viewer direction V, and the *x* axis onto the (x,y)-plane. The constants *A* and *B* are non-negative quantities and depend on the roughness parameter σ as follows:

$$\begin{array}{ccc} & & A=1-0.5\;\sigma^{2}{\left(\sigma^{2}+0.33\right)}^{-1}\\ & & B=0.45\sigma^{2}{\left(\sigma^{2}+0.09\right)}^{-1}. \end{array}$$

As a consequence of the chosen setup illustrated in Section 2.1, $\theta_{i}=\theta_{r}=\alpha =\beta$, and we can simply call it θ. Hence, adding the attenuation factor $1/r^{2}$ as proposed in [23], the Oren–Nayar brightness equation is as follows:

(14) $$I\left(x\right)=\frac{A\cos\left(\theta \right)+B\sin^{2}\left(\theta \right)}{r^{2}}.$$

Note that when σ=0, then A=1 and B=0, so the ON-model goes back to the L-model. Since r=fu(x), and cos(θ)=N(x)·ω(S(x)), from which $\sin^{2}\left(\theta \right)=1-{\left(N\left(x\right)\cdot \omega \left(S\left(x\right)\right)\right)}^{2}$, then the brightness Equation (14) becomes:

(15) $$\begin{array}{ccc} I\left(x\right)f^{2}u{\left(x\right)}^{2} & = & \frac{Au(x)Q(x)}{\sqrt{f^{2}{\left|\nabla u\right|}^{2}+{\left(\nabla u\cdot x\right)}^{2}+u{\left(x\right)}^{2}Q{\left(x\right)}^{2}}}\;\\ \; & \; & +B\left(1-\frac{u{\left(x\right)}^{2}Q{\left(x\right)}^{2}}{f^{2}{\left|\nabla u\right|}^{2}+{\left(\nabla u\cdot x\right)}^{2}+u{\left(x\right)}^{2}Q{\left(x\right)}^{2}}\right), \end{array}$$

where Q(x) is defined as in (9). By using the function *W* defined in (10), and the change of variable v(x)=lnu(x) (which is possible since $u\left(x\right)>0\;\forall x\in \bar{\Omega }$), we obtain the following HJ equation in *v*:

(16) $$-e^{-2v(x)}+I\left(x\right)f^{2}\frac{\left(W(x,\nabla v(x))+1\right)}{A\sqrt{W(x,\nabla v(x))+1}+BW\left(x,\nabla v\left(x\right)\right)}=0,$$

and we associate with it the following corresponding Hamiltonian

(17) $$\;H^{ON}\left(x,s,p\right):=-e^{-2s}+I\left(x\right)\;f^{2}\left[\frac{\left(W(x,p)+1\right)}{A\;\sqrt{W(x,p)+1}+BW\left(x,p\right)}\;\right].$$

Note that the function W(x,p), defined in (10), is the same function used in the L-model, which is a useful tool for the unified approach of different reflectance models.

#### 2.2.3. Phong Model

The Phong model (PH-model) [20] belongs to the category of specular reflectance models. The law of specular reflection states that the angle of reflection of a ray equals the angle of incidence, and that the incident direction, the surface normal and the reflected direction are coplanar. The PH-model considers a specular term in the definition of the function I(x):

(18) $$I\left(x\right)=k_{A}I_{A}\left(x\right)+k_{D}\gamma_{D}\left(x\right)\left(\cos\theta_{i}\right)+k_{S}\gamma_{S}\left(x\right){\left(\cos\theta_{s}\right)}^{\alpha },$$

where $I_{A}\left(x\right)$ is the ambient light component and $k_{A}$ the percentages of this component, the diffuse component is defined as in the L-model, with $k_{D}$ indicating the percentages of this component, and the third addend represents the specular light component, with $k_{S}$ indicating the percentages of it. This last term is described as a power of the cosine of the angle $\theta_{s}$ between the unit vectors V and R(x), with R indicating the reflection of the light ω on the surface; $\gamma_{S}\left(x\right)$ denotes the specular albedo, and α represents the characteristics of specular reflection of a material. We assume $k_{A}+k_{d}+k_{S}=1$, and that α is an integer greater or equal to 1 (in [20], Phong assumes α∈[1,10]). Under the chosen setup, the direction of the light source and that of the viewer are the same; therefore, $\theta_{s}=2\theta_{i}$ (see [25]). By setting the diffuse and specular albedo to 1 and adding the light attenuation term, we can arrive at the following non-linear PDE associated with the brightness Equation (18):

(19) $$\;I\left(x\right)=k_{A}I_{A}\left(x\right)+\frac{1}{f^{2}u{\left(x\right)}^{2}}\left(k_{D}\frac{u(x)Q(x)}{\left|n(x)\right|}+k_{S}{\left(2\frac{u^{2}Q{\left(x\right)}^{2}}{{\left|n\left(x\right)\right|}^{2}}-1\right)}^{\alpha }\right),$$

where Q(x) is defined as in (9), |n(x)| as in (6), $\cos\theta_{i}=N\left(x\right)\cdot \omega \left(S\left(x\right)\right)$ and $\cos\theta_{s}=\cos2\theta_{i}=2{\left(\cos\theta_{i}\right)}^{2}-1=2{\left(N\left(x\right)\cdot \omega \left(S\left(x\right)\right)\right)}^{2}-1$. By the same change of variable v(x)=lnu(x), using the function W(x,∇v(x)) defined in (10), we can obtain the following HJ equation in *v*:

(20) $$\begin{array}{ccc} \; & \; & \;-e^{-2v(x)}\;\\ \; & \; & \;+\left(I\left(x\right)-k_{A}I_{A}\left(x\right)\right)f^{2}\frac{\sqrt{W(x,\nabla v(x))+1}}{k_{D}+k_{S}\sqrt{W(x,\nabla v(x))+1}\;{\left(\frac{1-W(x,\nabla v(x))}{1+W(x,\nabla v(x))}\right)}^{\alpha }}=0. \end{array}$$

A different equation was derived in [25], defining an alternative function *W*, which, however, is not useful for our purpose of a unified formulation.

As stated in [25], since the term $\left(\frac{1-W(x,p)}{1+W(x,p)}\right)$ represents the cosine of the specular term, in the PH-model, this cosine is replaced by zero if $\cos\theta_{s}=\left(\frac{1-W(x,p)}{1+W(x,p)}\right)<0$. Hence, we can associate to (20) the following Hamiltonian:

(21) $$\begin{array}{ccc} & & \;H^{PH}\left(x,s,p\right):=\\ & & \;\left\{\begin{array}{c} -e^{-2s}+\left(I\left(x\right)-k_{A}I_{A}\left(x\right)\right)f^{2}\frac{\sqrt{W(x,p)+1}}{k_{D}}\\ \;\mathrm{if}\;W(x,p)\geq 1,\\ -e^{-2s}+\left(I\left(x\right)-k_{A}I_{A}\left(x\right)\right)f^{2}\frac{\sqrt{W(x,p)+1}}{k_{D}+k_{S}\sqrt{W(x,p)+1}\;{\left(\frac{1-W(x,p)}{1+W(x,p)}\right)}^{\alpha }}\\ \;\mathrm{if}\;0\leq W(x,p)<1. \end{array}\right. \end{array}$$

Note that the Phong model reduces to the Lambertian one (up to a constant) if $k_{S}=0$, i.e., the specular component vanishes.

#### 2.2.4. Blinn–Phong Model

In 1977, Blinn [21] proposed a modification of the Phong model by introducing an intermediate vector H, which bisects the angle between the unit vectors V and ω. If the surface is a perfect mirror, the light reaches the viewer only if the surface normal N is pointed halfway between the viewer direction V and the light source direction ω. The direction of maximum highlight is denoted by $H=\frac{\omega +V}{\left|\omega +V\right|}$. For less than perfect mirrors, the specular component falls off slowly as the normal direction moves away from the specular direction. The cosine of the angle between H and N is used as a measure of the distance of a particular surface from the maximum specular direction. The degree of sharpness of the highlights is adjusted by taking this cosine to some power (in [21], it is stated that this power is typically 50 or 60).

For the Blinn–Phong model (BP-model), the brightness equation is as follows:

(22) $$I\left(x\right)=k_{A}I_{A}\left(x\right)+k_{D}\gamma_{D}\left(x\right)\left(\cos\theta_{i}\right)+k_{S}\gamma_{S}\left(x\right){\left(\cos\delta \right)}^{c},$$

where δ is the angle between H and the unit normal N, and *c* measures the shininess of the surface (we assume c≥1). Using the setup introduced in Section 2.1, the directions of the light source and the viewer are the same. As already carried out for the PH-model, we set $\gamma_{D}\left(x\right)=\gamma_{S}\left(x\right)=1$ and we add the light attenuation term as performed for the previous models. In this way, Equation (22) becomes the following:

$$I\left(x\right)=k_{A}I_{A}\left(x\right)+\frac{1}{r^{2}}k_{D}\left(N\left(x\right)\cdot \omega \left(S\left(x\right)\right)\right)+k_{S}{\left(N\left(x\right)\cdot \omega \left(S\left(x\right)\right)\right)}^{c},$$

since $\cos\theta_{i}=N\left(x\right)\cdot \omega \left(S\left(x\right)\right)$ and cosδ=N(x)·ω(S(x)) due to the chosen setup. Using the definition of the unit normal N(x), thanks to (5) and (6), the function W(x,∇v(x)) as defined in (10), Q(x) as defined in (9), and the same change in variable adopted before, i.e., v(x)=lnu(x), we obtain the following HJ equation in the new variable *v* (see [29] for more details):

(23) $$-e^{-2v(x)}+\left(I\left(x\right)-k_{A}I_{A}\left(x\right)\right)f^{2}\frac{{\left(W\left(x,\nabla v\left(x\right)\right)+1\right)}^{c/2}}{k_{D}{\left(W\left(x,\nabla v\left(x\right)\right)+1\right)}^{\frac{c-1}{2}}+k_{S}}=0,$$

with which we associate the Hamiltonian

(24) $$H^{BP}\left(x,s,p\right):=-e^{-2s}+\left(I\left(x\right)-k_{A}I_{A}\left(x\right)\right)f^{2}\frac{{\left(W\left(x,p\right)+1\right)}^{c/2}}{k_{D}{\left(W\left(x,p\right)+1\right)}^{\frac{c-1}{2}}+k_{S}}.$$

**Remark** **1.** *As already observed for the PH-model, if $k_{S}=0$, the BP-model also reduces to the Lambertian one, up to a constant.*

### 2.3. Reflectance Models in a Unified Formulation

The four different reflectance models presented in Section 2.2 lead to a HJ equation of the following form:

(25) H(x,v(x),∇v(x))=0,∀x∈Ω,

where v(x) is related to the original unknown u(x), which represents the surface height, thanks to the change of variable v(x)=lnu(x). The Hamiltonian *H* has a peculiar explicit formulation for each of the four models, given by (12), (17), (21) and (24), respectively. Nevertheless, looking at the four Hamiltonians, we can note that (25) can be rewritten separating a term depending only on v(x) and a Hamiltonian which depends only on (x,∇v(x)) as follows:

(26) $$-e^{-2v}+H^{M}\left(x,\nabla v\left(x\right)\right)=0$$

where *M* indicates the acronym of the four models (M=L,ON,PH,BP). Since the function W(x,p) adopted is the same for all four cases, then $H^{M}\left(x,p\right)$ corresponds for each one to the following:

*Lambertian model*

(27) $$\begin{array}{cc} \; & H^{L}\left(x,p\right):=I\left(x\right)f^{2}F_{L}\left(W\left(x,p\right)\right), \end{array}$$

where

(28) $$\begin{array}{cc} \; & F_{L}\left(s\right)=\sqrt{s+1}. \end{array}$$

*Oren–Nayar model*

(29) $$\begin{array}{cc} \; & H^{ON}\left(x,p\right)=I\left(x\right)f^{2}F_{ON}\left(W\left(x,p\right)\right), \end{array}$$

where

(30) $$\begin{array}{cc} \; & F_{ON}\left(s\right)=\frac{s+1}{A\sqrt{s+1}+Bs}. \end{array}$$

*Phong model*

(31) $$\begin{array}{cc} \; & H^{PH}\left(x,p\right)=\left(I\left(x\right)-k_{A}I_{A}\left(x\right)\right)f^{2}F_{PH}\left(W\left(x,p\right)\right), \end{array}$$

where

(32) $$\begin{array}{cc} \; & F_{PH}\left(s\right)=\left\{\begin{array}{cc} \frac{\sqrt{s+1}}{k_{D}} & \mathrm{if}\;s\geq 1\\ \frac{{\left(s+1\right)}^{\alpha +1/2}}{k_{D}{\left(s+1\right)}^{\alpha }+k_{S}{\left(s+1\right)}^{1/2}{\left(1-s\right)}^{\alpha }} & \mathrm{if}\;0\leq s<1. \end{array}\right. \end{array}$$

*Blinn–Phong model*

(33) $$\begin{array}{cc} \; & H^{BP}\left(x,p\right)=\left(I\left(x\right)-k_{A}I_{A}\left(x\right)\right)f^{2}F_{BP}\left(W\left(x,p\right)\right), \end{array}$$

where

(34) $$\begin{array}{cc} \; & F_{BP}\left(s\right)=\frac{{\left(s+1\right)}^{c/2}}{k_{D}{\left(s+1\right)}^{\frac{c-1}{2}}+k_{S}}. \end{array}$$

In [29], some useful bounds on the function *W* were proved. In particular, the authors showed the following:

$$f^{2}{\left|p\right|}^{2}\leq W\left(x,p\right)\leq \bar{C}{\left|p\right|}^{2}$$

where the constant $\bar{C}$ is independent of $\left(x,p\right)\in \bar{\Omega }\times R^{2}$. These bounds help to prove our convergence result. Some properties of the four functions $F_{M}$ can also be proved (see Lemma 4 in [29]).

### 2.4. Viscosity Solutions of Hamilton–Jacobi Equations

Let us recall in this section the definition and basic properties of viscosity solutions in order to study the general Hamiltonian (25) in the case of our reflectance models (the reader can refer to [9,11] for more details on this theory). As already stated in the introduction, a HJ equation such as (25), in general, does not admit a classical solution, and we need to look for a proper notion of a weak solution, for example, in the context of viscosity solutions. However, if a classical solution to the HJ equation exists, it coincides with the viscosity one. Tipically, viscosity solutions are Lipschitz continuous functions, which can be obtained via the method of vanishing viscosity, i.e., as a limit of regular solutions to second-order problems.

We can now give the definition of a viscosity subsolution, supersolution and solution.

**Definition** **1.** *A viscosity supersolution of (25) on $\Omega \subset R^{2}$ is a lower semicontinuous (LSC) function, v:Ω→R, s.t. for any $\psi \in C^{1}\left(\Omega \right)$, if $x_{0}\in \Omega$ is a local minimum point of v−ψ, then*

$$H(x_{0},v\left(x_{0}\right),\nabla \psi \left(x_{0}\right))\geq 0.$$

*A viscosity subsolution of (25) on $\Omega \subset R^{2}$ is a upper semicontinuous (USC) function, v:Ω→R, s.t. for any $\psi \in C^{1}\left(\Omega \right)$, if $x_{0}\in \Omega$ is a local maximum point of v−ψ, then*

$$H(x_{0},v\left(x_{0}\right),\nabla \psi \left(x_{0}\right))\leq 0.$$

*Finally, v is a viscosity solution of (25) if it is both a viscosity super- and subsolution.*

To prove the convergence of a numerical scheme using the Barles–Souganidis approach [32], we need the *Maximum Principle for discontinuous viscosity solution*, which requires the formulation in a weak (viscosity) sense of the boundary conditions for the PDE to be approximated. Of course, this requirement is in addition to the consistency, monotonicity and stability properties of the numerical scheme. Hence, motivated by this remark, we need to introduce the notion of boundary conditions in a weak (viscosity) sense, which means we have to incorporate the boundary condition into the definition. To gain a better understanding, instead of considering, for example, the Dirichlet boundary condition in a pointwise sense associated with the HJ Equation (25), i.e., v(x)=g(x)∀x∈∂Ω, we introduce the operator $B:\partial \Omega \times R\times R^{2}\to R$, which represents the boundary condition, and in the case of the Dirichlet boundary condition, it is chosen as B(x,s,p)=s−g(x). Let us consider a more general boundary value problem of the following form:

(35) $$\left\{\begin{array}{cc} H(x,v(x),\nabla v(x))=0, & \forall x\in \Omega ,\\ B(x,v(x),\nabla v(x))=0, & \forall x\in \partial \Omega , \end{array}\right.$$

with the operator $B:\partial \Omega \times R\times R^{2}\to R$. We now introduce the notion of (discontinuous) viscosity solution for (35).

**Definition** **2.** *A viscosity supersolution of (35) is a LSC function v in $\bar{\Omega }$ s.t. for any $\psi \in C^{1}\left(\bar{\Omega }\right)$, if $x_{0}$ is a local minimum point of v−ψ, then*

$$\begin{array}{ccc} \; & H(x_{0},v\left(x_{0}\right),\nabla \psi \left(x_{0}\right))\geq 0, & if\;x\in \Omega ,\\ \; & \max[H\left(x_{0},v\left(x_{0}\right),\nabla \psi \left(x_{0}\right)\right),\;B\left(x_{0},v\left(x_{0}\right),\nabla \psi \left(x_{0}\right)\right)]\geq 0, & if\;x\in \partial \Omega . \end{array}$$

*A viscosity subsolution of (35) is a USC function v in $\bar{\Omega }$ s.t. for any $\psi \in C^{1}\left(\bar{\Omega }\right)$, if $x_{0}$ is a local maximum point of v−ψ, then*

$$\begin{array}{ccc} \; & H(x_{0},v\left(x_{0}\right),\nabla \psi \left(x_{0}\right))\leq 0, & if\;x\in \Omega ,\\ \; & \min[H\left(x_{0},v\left(x_{0}\right),\nabla \psi \left(x_{0}\right)\right),\;B\left(x_{0},v\left(x_{0}\right),\nabla \psi \left(x_{0}\right)\right)]\leq 0, & if\;x\in \partial \Omega . \end{array}$$

*Finally, v is a viscosity solution of (35) if it is both a viscosity super- and subsolution.*

We are now ready to state the *Maximum Principle for discontinuous viscosity solutions*. We first consider the case of a Dirichlet boundary condition (see [9]).

**Theorem** **1.** *Let $\Omega \subset R^{2}$ be bounded with ∂Ω of class $W^{2,\infty }$, g:∂Ω→R a continuous function and $H:\Omega \times R\times R^{2}\to R$ satisfying the following two conditions:*

(36) $$\begin{array}{c} (H1).\;\mathrm{For}\;\mathrm{any}\;R>0,\;\exists \;\gamma_{R}>0\;\mathrm{such}\;\mathrm{that}\;H\left(x,v,p\right)-H\left(x,u,p\right)\geq \gamma_{R}\left(v-u\right)\\ \;\mathrm{for}\;\mathrm{all}\;x\in \Omega ,\;-R\leq u\leq v\leq R\;\mathrm{and}\;p\in R^{2}, \end{array}$$

(37) $$\begin{array}{c} (H2).\;\left|H\left(x,v,p\right)-H\left(y,v,p\right)\right|\leq m_{R}(|x-y|(1+\left|p|)\right)\;\mathrm{for}\;\mathrm{all}\;x,y\in \Omega ,\;\\ -R\leq v\leq R,\;p\in R^{2}\;\mathrm{where}\;m_{R}\left(t\right)\to 0\;\mathrm{when}\;t\to 0. \end{array}$$

*Moreover, let us assume that there exists a neighborhood *Γ* of ∂Ω in $R^{2}$, such that*

(38) $$\begin{array}{c} (H3).\;\forall R\in \left(0,+\infty \right)\;\;\exists \;\;m_{R}\left(t\right)\to 0\;\mathrm{when}\;t\to 0\;\mathrm{such}\;\mathrm{that}\\ \left|H\left(x,v,p\right)-H\left(x,v,q\right)\right|\leq m_{R}(|p-q|)),\;\forall x\in \Gamma ,\;v\in [-R,R],\;\;p,q\in R^{2}, \end{array}$$

(39) $$\begin{array}{c} (H4).\;\forall R\in \left(0,+\infty \right)\;\;\exists \;\;C_{R}>0\;\mathrm{such}\;\mathrm{that}\;H(x,v,p+\lambda n(x))\leq 0\\ \Rightarrow \lambda \leq C_{R}\left(1+|p|\right),\;\forall \left(x,v,p\right)\in \Gamma \times \left[-R,R\right]\times R^{2}, \end{array}$$

(40) $$\begin{array}{c} (H5).\;\forall R_{1},R_{2}\in R^{+},\;H\left(x,v,p-\lambda n\left(x\right)\right)\to +\infty \;\lambda \to +\infty ,\;\\ \;\mathrm{uniformly}\;\mathrm{for}\;\left(x,v,p\right)\in \Gamma \times \left[-R_{1},R_{2}\right]\times B\left(0,R_{2}\right). \end{array}$$

*Let v be a USC function in $\bar{\Omega }$, subsolution of (35) (u is a LSC function in $\bar{\Omega }$, supersolution of (35), respectively) with B(x,s,p)=s−g(x). Then, v≤u in Ω.*

This theorem implies the uniqueness of the viscosity solution in $\bar{\Omega }$, i.e., if $v_{1},v_{2}\in C\left(\bar{\Omega }\right)$ are two viscosity solutions of (35), then $v_{1}=v_{2}$ in $\bar{\Omega }$. If we want to consider different boundary conditions and validate the Maximum Principle, it is sufficient to choose the following:

− B(x,s,p)=N(x)·p−g(x) for the Neumann boundary condition, assuming *H* is continuous and satisfying the hypothesis (H1)–(H3) reported in Equations (36)–(38).
− B(x,s,p)=s−g(x) with g(x)≡−∞ for the state constraints condition, assuming *H* is continuous and satisfying the hypothesis (H1)–(H4) reported in Equations (36)–(39).

Defining

(41) $$J\left(x\right):=\left\{\begin{array}{cc} I(x)\; & \mathrm{if}\;H=H^{L},H^{ON},\\ I\left(x\right)-k_{A}I_{A}\left(x\right) & \mathrm{if}\;H=H^{PH},H^{BP}, \end{array}\right.$$

and assuming that J(x) is a Lipschitz continuous function in $\bar{\Omega }$ and there exists a neighborhood Γ of ∂Ω, such that J(x)≥δ>0 in Γ, in [29], the authors validated the assumptions for the Maximum Principle related to the four Hamiltonians, $H^{L},H^{ON},H^{PH}$, and $H^{BP}$ defined in (12), (17), (21) and (24), respectively, arriving to prove the well-posedness of the perspective SfS problems.

**Remark** **2.** *Without the introduction of the attenuation term and/or under a different setup, e.g., in the context of orthographic projection, the assumption (36) would not be satisfied; hence, uniqueness would not be guaranteed.*

## 3. Convergence Result

Following [32], we now establish necessary notions for the convergence of numerical schemes in the viscosity sense: consistency, monotonicity and stability. Afterwards, we bring in the convergence theorem [32], with which we can prove the convergence of our scheme.

To approximate (25), we consider a scheme of the following form:

(42) $$S\left(\rho ,x,u^{\rho }\left(x\right),u^{\rho }\right)=0\;\;\mathrm{in}\;\bar{\Omega },$$

where $S:R^{+}\times \bar{\Omega }\;\times R\times B\left(\bar{\Omega }\right)\to R$ is locally bounded, $R^{+}\equiv \left[0,\infty \right)$, ρ is a discretization parameter and $B(\bar{\Omega })$ denotes the space of bounded functions defined on $\bar{\Omega }$.

**Definition** **3** (**Consistency**)**.** *The scheme defined by (42) is assumed to be consistent, provided that both the following conditions are satisfied:*

*For all $x_{0}\in \bar{\Omega }$ and $\psi \in C^{1}\left(\bar{\Omega }\right)$*

(43) $$\underset{\rho \to 0\;,\;x\to x_{0},\;\xi \to 0}{lim inf}\frac{S\left(\rho ,x,\psi \left(x\right)+\xi ,\psi +\xi \right)}{\rho }\geq H_{*}\left(x_{0},\psi \left(x_{0}\right),D\psi \left(x_{0}\right)\right)$$

*and*

(44) $$\underset{\rho \to 0\;,\;x\to x_{0}\;,\;\xi \to 0}{lim sup}\frac{S\left(\rho ,x,\psi \left(x\right)+\xi ,\psi +\xi \right)}{\rho }\leq H^{*}\left(x_{0},\psi \left(x_{0}\right),D\psi \left(x_{0}\right)\right)\;,$$

where $H_{*}$ and $H^{*}$ denote viscosity sub- and supersolution, respectively, defined in the previous section.

**Definition** **4** (**Monotonicity**)**.** *The scheme S in (42) is assumed to be monotone, provided that the following condition is fulfilled*

(45) S(ρ,x,t,u)≤S(ρ,x,t,v)

*if u≥v for all ρ≥0, $x\in \bar{\Omega }$, t∈R and $u,v\in B(\bar{\Omega })$.*

**Definition** **5** (Stability). *The scheme S in (42) is called stable if for all ρ>0, there exists a solution $u^{\rho }\in B\left(\bar{\Omega }\right)$ of (42) with a bound that is independent of ρ.*

Based on these definitions, the convergence theorem in the viscosity framework is elaborated by the following fundamental result (cf. [32]):

**Theorem** **2** (**Convergence**)**.** *If an HJ equation such as (25) has a strong uniqueness property and if the scheme S as in (42) is consistent, monotone and stable, then the solution $u^{\rho }$ of S converges locally uniformly to the unique viscosity solution u of the original problem (25) as ρ→0.*

### 3.1. Numerical Discretization

For the sake of simplicity, we stick to the 1-D version of (26) since this suffices to perceive the methodology. The 1-D model of (26) reads as follows:

(46) $$-e^{-2v}+H^{M}\left(x,v_{x}\left(x\right)\right)=0$$

where *M* is the acronym of the models (M=L,ON,PH,BP). For each model, the corresponding $H^{M}\left(x,v_{x}\left(x\right)\right)$ uses the 1D version of (9), (10) and the various functions $F_{M}$.

Let Δx>0 be the spatial step size, and we denote by N:=N(Δx) the number of grid points $x_{i}$, i=1,…,N. We further denote the approximate value of *v* at the *i*-th grid point $x_{i}$ by $w_{i}$ and define $\psi_{i}\left(w\right)$ as the following:

(47) $$\psi_{i}\left(w\right):=\max\left(0,-D^{+}w,D^{-}w\right),\;i=1,\ldots ,N\;,\;$$

where $w\;=\;\left(w_{1},\ldots ,w_{N}\right)$, cf. [34]. In addition, for consistency, the approximate spatial derivative is given by the following:

(48) $$v_{x}\left(x_{i}\right)\approx {\hat{w}}_{x,i}:=\psi_{i}\left(w\right)=\left\{\begin{array}{cc} D^{+}w=\frac{w_{i+1}-w_{i}}{\Delta x}<0 & \mathrm{if}\;-D^{+}w\;\mathrm{is}\;\mathrm{chosen}\;\\ D^{-}w=\frac{w_{i}-w_{i-1}}{\Delta x}>0 & \mathrm{if}\;D^{-}w\;\mathrm{is}\;\mathrm{chosen}\;. \end{array}\right.$$

In order to discretize (46), we introduce the method of artificial time, and we make use of the Euler forward in time scheme in addition to (48), obtaining at each grid point the following approximation scheme:

(49) $$-e^{-2w_{i}^{n}}+\frac{w_{i}^{n+1}-w_{i}^{n}}{\Delta t}+H^{M}\left(x_{i},{\hat{w}}_{x,i}\right)=0\;,$$

which we can rewrite to obtain the following update rule:

(50) $$w_{i}^{n+1}=w_{i}^{n}+\Delta t\;e^{-2w_{i}^{n}}-\Delta t\;H^{M}\left(x_{i},{\hat{w}}_{x,i}\right)=:G\left(x_{i},w_{i},{\hat{w}}_{x,i}\right)\;.$$

For simplicity, here, we also assume that the iteration level used for the discrete representations of $v_{x}$ is always the actual level *n*. This also holds for the source term, i.e., $e^{-2v}\approx e^{-2w_{i}^{n}}$.

### 3.2. Proof of the Convergence Result

We now state the main result of this paper:

**Theorem** **3.** *Let us assume that the scheme given in (50) satisfies the assumptions of the Theorem 2 and the HJ equation in (46) admits a strong uniqueness property. Then, the solution of (50) converges locally uniformly to the unique continuous viscosity solution of (46).*

**Proof.**  We have to validate the assumptions of strong uniqueness, consistency, monotonicity and stability.

Regarding the strong uniqueness for (46), Propositions 9 and 11 in [29] already validated the required conditions. In addition, the scheme (50) is consistent with the HJ Equation (46) since this is directly followed by the construction. Moreover, by [35], the stability holds when the scheme is monotone. Hence, the verification of the monotonicity remains. To this end, by [35], it is sufficient to show that G in (50) is a non-decreasing function, which holds if and only if:

(51) $$\left(i\right)\;\frac{\partial G}{\partial w_{i-1}}\geq 0,\;\left(ii\right)\;\frac{\partial G}{\partial w_{i}}\geq 0,\;\left(iii\right)\;\frac{\partial G}{\partial w_{i+1}}\geq 0\;.$$

Due to analogy between case (i) and (iii), we proceed in the following order: case (i)→(iii)→(ii).

**Case (i).**

In view of (47) and (48), there is a contribution to the following case (i) only when the third argument in (47) is taken. Taking a partial derivative of (50) with respect to $w_{i-1}$ yields the following:

(52) $$\frac{\partial G}{\partial w_{i-1}}=-\Delta t\;\frac{\partial H^{M}}{\partial w_{i-1}}=\left\{\begin{array}{c} -\Delta t\left(If^{2}\right)\frac{\partial F_{L,ON}}{\partial w_{i-1}}\\ -\Delta t\left(I-I_{a}k_{a}\right)f^{2}\frac{\partial F_{PH,BP}}{\partial w_{i-1}} \end{array}\right.$$

Note that $-\Delta t(If^{2})$ or $-\Delta t\left(I-I_{a}k_{a}\right)f^{2}$ are both negative quantities. Therefore, in order to obtain $\frac{\partial G}{\partial w_{i-1}}\geq 0$, it is necessary to have $\frac{\partial F_{M}}{\partial w_{i-1}}\leq 0$.

At this point, we need to distinguish the four cases. Note that for construction and for Lemma 3 of [29], the function $W(x,v_{x})\geq 0$. In the following, we omit the variable dependencies for brevity.

*Lambertian case ($F_{L}\left(W\right)=\sqrt{W+1}$)*

(53) $$\frac{\partial F_{L}}{\partial w_{i-1}}=\frac{1}{2\sqrt{W+1}}\cdot \frac{\partial W}{\partial w_{i-1}}=\frac{1}{2\sqrt{W+1}}\cdot \frac{2\left(f^{2}+x^{2}\right)\left(w_{i}-w_{i-1}\right)\left(-1\right)}{Q^{2}{\left(\Delta x\right)}^{2}}<0$$

Hence, $\frac{\partial G}{\partial w_{i-1}}\geq 0$ for the Lambertian case.

*Oren–Nayar case ($F_{ON}\left(W\right)=\frac{W+1}{A\sqrt{W+1}+BW}$)*

(54) $$\frac{\partial F_{ON}}{\partial w_{i-1}}=\frac{\frac{\partial W}{\partial w_{i-1}}\left(A\sqrt{W+1}+BW\right)-\left(W+1\right)\cdot \frac{\partial }{\partial w_{i-1}}\left(A\sqrt{W+1}+BW\right)}{{\left(A\sqrt{W+1}+BW\right)}^{2}}$$

Since the denominator is always positive, we focus only on the numerator:

(55) $$\begin{array}{c} \begin{array}{cc} & \frac{\partial W}{\partial w_{i-1}}\left(A\sqrt{W+1}+BW\right)-\left(W+1\right)\cdot \frac{\partial }{\partial w_{i-1}}\left(A\sqrt{W+1}+BW\right)\\ = & \frac{\partial W}{\partial w_{i-1}}\left(A\sqrt{W+1}+BW\right)-\left(W+1\right)\cdot \left(\frac{A}{2\sqrt{W+1}}\frac{\partial W}{\partial w_{i-1}}+B\frac{\partial W}{\partial w_{i-1}}\right)\\ = & \underset{<0}{\underset{︸}{\frac{\partial W}{\partial w_{i-1}}}}\underset{\geq 0\;\mathrm{if}\;\frac{A}{2}>B}{\underset{︸}{\left[\sqrt{W+1}\left(\frac{A}{2}\right)-B\right]}} \end{array} \end{array}$$

Hence, $\frac{\partial F_{ON}}{\partial w_{i-1}}\leq 0$ if $\frac{A}{2}>B$. As a consequence, $\frac{\partial G}{\partial w_{i-1}}\geq 0$ for the Oren–Nayar case, under the assumption $\frac{A}{2}>B$.

*Blinn–Phong case ($F_{BP}\left(W\right)=\frac{{\left(W+1\right)}^{c/2}}{k_{D}{\left(W+1\right)}^{\frac{c-1}{2}}+k_{S}}$, with c≥1)*

(56) $$\begin{array}{cc} \; & \frac{\partial F_{BP}}{\partial w_{i-1}}=\\ \; & \frac{\frac{\partial {\left(W+1\right)}^{c/2}}{\partial w_{i-1}}\cdot \left(k_{D}{\left(W+1\right)}^{\frac{c-1}{2}}+k_{S}\right)-{\left(W+1\right)}^{c/2}\cdot \frac{\partial (k_{D}{\left(W+1\right)}^{\frac{c-1}{2}}+k_{S})}{\partial w_{i-1}}}{{\left(k_{D}{\left(W+1\right)}^{\frac{c-1}{2}}+k_{S}\right)}^{2}} \end{array}$$

Since the denominator is always greater than zero, we continue focusing only on the numerator.

(57) $$\begin{array}{cc} & \frac{\partial {\left(W+1\right)}^{c/2}}{\partial w_{i-1}}\cdot \left(k_{D}{\left(W+1\right)}^{\frac{c-1}{2}}+k_{S}\right)-{\left(W+1\right)}^{c/2}\cdot \frac{\partial (k_{D}{\left(W+1\right)}^{\frac{c-1}{2}}+k_{S})}{\partial w_{i-1}}\\ = & \underset{>0}{\underset{︸}{\frac{c}{2}}}\underset{>0}{\underset{︸}{{\left(W+1\right)}^{\frac{c}{2}-1}}}\underset{<0}{\underset{︸}{\frac{\partial W}{\partial w_{i-1}}}}\cdot \underset{>0}{\underset{︸}{\left(k_{D}{\left(W+1\right)}^{\frac{c-1}{2}}+k_{S}\right)}}\\ & -\underset{<0}{\underset{︸}{{\left(W+1\right)}^{\frac{c}{2}}}}\cdot \underset{>0}{\underset{︸}{\left(k_{D}\frac{c-1}{2}{\left(W+1\right)}^{\frac{c-1}{2}-1}\right)}}\cdot \underset{<0}{\underset{︸}{\frac{\partial W}{\partial w_{i-1}}}}\\ = & \frac{\partial W}{\partial w_{i-1}}{\left(W+1\right)}^{\frac{c}{2}-1}\left[k_{D}{\left(W+1\right)}^{\frac{c-1}{2}}\left(\frac{c}{2}-\frac{c-1}{2}\right)+\frac{c}{2}k_{S}\right]\\ = & \underset{<0}{\underset{︸}{\frac{\partial W}{\partial w_{i-1}}}}\left[\underset{>0}{\underset{︸}{k_{D}{\left(W+1\right)}^{\frac{c}{2}-1+\frac{c-1}{2}}\cdot \frac{1}{2}}}+\underset{>0}{\underset{︸}{{\left(W+1\right)}^{\frac{c}{2}-1}\cdot \frac{c}{2}k_{S}}}\right]<0 \end{array}$$

Hence, $\frac{\partial F_{BP}}{\partial w_{i-1}}<0\Rightarrow \frac{\partial G}{\partial w_{i-1}}\geq 0$ for the Blinn–Phong case.

*Phong case* $\left(F_{PH}\left(W\right)=\left\{\begin{array}{cc} \frac{\sqrt{W+1}}{k_{D}} & \mathrm{if}\;W\;\geq \;1\\ \frac{{\left(W+1\right)}^{\alpha +1/2}}{k_{D}{\left(W+1\right)}^{\alpha }+k_{S}{\left(W+1\right)}^{1/2}{\left(1-W\right)}^{\alpha }} & \mathrm{if}\;0\;\leq \;W<1 \end{array}\right.\right)$

In the case of W≥1, we fall back on the Lambertian case, so it is already proven. In the case 0≤W<1

(58) $$\;\begin{array}{c} \frac{\partial F_{PH}}{\partial w_{i-1}}\\ \;\;\;\;\;\;\;\;=\left(\left(\alpha +\frac{1}{2}\right)\cdot {\left(W+1\right)}^{\alpha +\frac{1}{2}-1}\cdot \frac{\partial W}{\partial w_{i-1}}\cdot \left[k_{D}{\left(W+1\right)}^{\alpha }+k_{S}{\left(W+1\right)}^{1/2}{\left(1-W\right)}^{\alpha }\right]\right.\\ \;\;\;\;\;\;\;\;\;\;\;\;\;\;\;\;\;\;\;\;\;\;\;\;\;\;\;-\left.{\left(W+1\right)}^{\alpha +\frac{1}{2}}\cdot \frac{\partial (k_{D}{\left(W+1\right)}^{\alpha }+k_{S}{\left(W+1\right)}^{1/2}{\left(1-W\right)}^{\alpha })}{\partial w_{i-1}}\right)\\ \;\;\;\;\;\;\;\;\;\;\;\;\;\;\;\;\;\;\;\;\;\;\;\;\;\;\;\;\;\;\;\;\;\;\;\;\;\;\;\;\;\;\;\;\;\;\;\;\;\;\;\cdot \frac{1}{{\left[k_{D}{\left(W+1\right)}^{\alpha }+k_{S}{\left(W+1\right)}^{1/2}{\left(1-W\right)}^{\alpha }\right]}^{2}} \end{array}$$

Since the denominator is always greater than zero, we continue focusing only on the numerator.

(59) $$\begin{array}{ccc} & & \left(\alpha +\frac{1}{2}\right)\cdot {\left(W+1\right)}^{\alpha -\frac{1}{2}}\cdot \frac{\partial W}{\partial w_{i-1}}\cdot \left[k_{D}{\left(W+1\right)}^{\alpha }+k_{S}{\left(W+1\right)}^{1/2}{\left(1-W\right)}^{\alpha }\right]\\ & & -{\left(W+1\right)}^{\alpha +\frac{1}{2}}\cdot \left[k_{D}\;\alpha {\left(W+1\right)}^{\alpha -1}\cdot \frac{\partial W}{\partial w_{i-1}}+\frac{k_{S}}{2}{\left(W+1\right)}^{-1/2}\frac{\partial W}{\partial w_{i-1}}\cdot {\left(1-W\right)}^{\alpha }\right.\\ & & \left.-k_{S}{\left(W+1\right)}^{1/2}\cdot \alpha {\left(1-W\right)}^{\alpha -1}\cdot \frac{\partial W}{\partial w_{i-1}}\right]\\ & = & \left(\alpha +\frac{1}{2}\right)\cdot {\left(W+1\right)}^{\alpha -\frac{1}{2}}\cdot \frac{\partial W}{\partial w_{i-1}}\cdot \left[k_{D}{\left(W+1\right)}^{\alpha }+k_{S}{\left(W+1\right)}^{1/2}{\left(1-W\right)}^{\alpha }\right]\\ & & -{\left(W+1\right)}^{\alpha +\frac{1}{2}}\cdot \left[\frac{\partial W}{\partial w_{i-1}}(k_{D}\;\alpha {\left(W+1\right)}^{\alpha -1}+\frac{k_{S}}{2}{\left(W+1\right)}^{-1/2}\cdot {\left(1-W\right)}^{\alpha }\right.\\ & & \left.-k_{S}{\left(W+1\right)}^{1/2}\cdot \alpha {\left(1-W\right)}^{\alpha -1})\right]\\ & = & \left(\alpha +\frac{1}{2}\right)\cdot {\left(W+1\right)}^{\alpha -\frac{1}{2}}\cdot \frac{\partial W}{\partial w_{i-1}}\cdot \left[k_{D}{\left(W+1\right)}^{\alpha }+k_{S}{\left(W+1\right)}^{1/2}{\left(1-W\right)}^{\alpha }\right]\\ & & -{\left(W+1\right)}^{\alpha +\frac{1}{2}}\cdot \left[\frac{\partial W}{\partial w_{i-1}}\left({\left(W+1\right)}^{\alpha -1}\cdot \left(k_{D}\;\alpha +\frac{k_{S}}{2}{\left(1-W\right)}^{\alpha }{\left(W+1\right)}^{-(\alpha -1/2)}\right.\right.\right.\\ & & \left.\left.\left.-k_{S}\;\alpha {\left(1-W\right)}^{\alpha -1}{\left(W+1\right)}^{-\alpha +3/2}\right)\right)\right] \end{array}$$

$$\begin{array}{ccc} & = & \frac{\partial W}{\partial w_{i-1}}\cdot \left[k_{D}\;\left(\alpha +\frac{1}{2}\right)\cdot {\left(W+1\right)}^{2\alpha -\frac{1}{2}}+k_{S}\left(\alpha +\frac{1}{2}\right)\cdot {\left(W+1\right)}^{\alpha }{\left(1-W\right)}^{\alpha }\right.\\ & & \left.-k_{D}\;\alpha {\left(W+1\right)}^{2\alpha -\frac{1}{2}}-\frac{k_{S}}{2}{\left(1-W\right)}^{\alpha }{\left(W+1\right)}^{\alpha }+k_{S}\;\alpha {\left(1-W\right)}^{\alpha -1}{\left(W+1\right)}^{\alpha +1}\right]\\ & = & \underset{<0}{\underset{︸}{\frac{\partial W}{\partial w_{i-1}}}}\cdot \left[\underset{>0}{\underset{︸}{{\left(W+1\right)}^{2\alpha -\frac{1}{2}}}}\underset{\geq 0}{\underset{︸}{\left(k_{D}\;\left(\alpha +\frac{1}{2}\right)-k_{D}\;\alpha \right)}}\right.\\ & & \left.+\underset{>0}{\underset{︸}{{\left(W+1\right)}^{\alpha }}}\underset{>0}{\underset{︸}{{\left(1-W\right)}^{\alpha }}}\cdot \underset{\geq 0}{\underset{︸}{\left(k_{S}\;\alpha +\frac{k_{S}}{2}-\frac{k_{S}}{2}\right)}}+\underset{\geq 0}{\underset{︸}{k_{S}\;\alpha }}\underset{>0}{\underset{︸}{{\left(1-W\right)}^{\alpha -1}}}\underset{>0}{\underset{︸}{{\left(W+1\right)}^{\alpha +1}}}\right] \end{array}$$

Hence, we conclude that $\frac{\partial F_{PH}}{\partial w_{i-1}}\leq 0$, so $\frac{\partial G}{\partial w_{i-1}}\geq 0$ also for the Phong case.

**Case (iii).**

For the case (iii), there is a contribution only when the second argument in (47) is chosen. Taking partial derivative of (50) with respect to $w_{i+1}$ yields the following:

(60) $$\frac{\partial G}{\partial w_{i+1}}=-\Delta t\;\frac{\partial H^{M}}{\partial w_{i+1}}=\left\{\begin{array}{c} -\Delta t\left(If^{2}\right)\frac{\partial F_{L,ON}}{\partial w_{i+1}}\\ -\Delta t\left(I-I_{a}k_{a}\right)f^{2}\frac{\partial F_{PH,BP}}{\partial w_{i+1}} \end{array}\right.$$

As for case (i), note that $-\Delta t(If^{2})$ or $-\Delta t\left(I-I_{a}k_{a}\right)f^{2}$ are both negative quantities. Therefore, in order to obtain $\frac{\partial G}{\partial w_{i+1}}\geq 0$, it is necessary to show that $\frac{\partial F_{M}}{\partial w_{i+1}}\leq 0$.

We note that

(61) $$\frac{\partial W}{\partial w_{i+1}}=\frac{\left(2f^{2}+x^{2}\right)\left(w_{i+1}-w_{i}\right)\left(+1\right)}{Q^{2}\Delta x^{2}}<0\;.$$

Hence, the quantity $\frac{\partial W}{\partial w_{i+1}}$ is less than zero in this case, as it was $\frac{\partial W}{\partial w_{i-1}}$ in the case (i). As a consequence of this remark, the proof for this case (iii) is analogous to the previous case (i) for all four functions *F*, resulting in $\frac{\partial G}{\partial w_{i+1}}\geq 0$ for each model under the same assumptions.

**Case (ii).**

Taking partial derivatives of (50) with respect to $w_{i}$ results in the following:

(62) $$\begin{array}{ccc} \frac{\partial G}{\partial w_{i}}\; & = & \frac{\partial w_{i}}{\partial w_{i}}+\Delta t\;\frac{\partial e^{-2w_{i}}}{\partial w_{i}}-\Delta t\;\frac{\partial H^{M}}{\partial w_{i}}\;\\ \; & = & 1-2\Delta t\;e^{-2w_{i}}-\Delta t\;\frac{\partial H^{M}}{\partial w_{i}}.\; \end{array}$$

At this point, we need to distinguish the four models.

*Lambertian case* ($H^{L}=If^{2}F_{L}\left(W\right)=\left(If^{2}\right)\sqrt{W+1}$)

(63) $$\frac{\partial G}{\partial w_{i}}=1-2\Delta t\;e^{-2w_{i}}-\Delta tIf^{2}\left(\frac{2(f^{2}+x^{2})}{2\sqrt{W+1}Q^{2}}{\hat{w}}_{x,i}\frac{\partial {\hat{w}}_{x,i}}{\partial w_{i}}\right),$$

recalling that ${\hat{w}}_{x,i}:=\max\left(0,-D^{+}w,D^{-}w\right)$.

By definition of ${\hat{w}}_{x,i}$, we note that it is always, in any case, a non-negative quantity and

(64) $$\frac{\partial {\hat{w}}_{x,i}}{\partial w_{i}}=\left\{\begin{array}{cc} 0 & \mathrm{if}\;0\;\mathrm{is}\;\mathrm{chosen}\\ -\frac{1}{\Delta x}<0 & \mathrm{if}\;-D^{+}w\;\mathrm{is}\;\mathrm{chosen}\;\\ \frac{1}{\Delta x}>0 & \mathrm{if}\;D^{-}w\;\mathrm{is}\;\mathrm{chosen}\;. \end{array}\right.$$

Since a violation of case (ii) primarily occurs due to the quantity $\left(f^{2}+x^{2}\right){\hat{w}}_{x,i}$ in the last term of (63), we incorporate a worst case estimate as follows:

(65) $${\hat{w}}_{x,i}\leq \max\left(\left|{\hat{w}}_{x,i}\right|\right)=:{\left|\hat{w}\right|}_{\max}\;,$$

where the maximum is taken over all possible cases in (48). It follows

(66) $$\begin{array}{cc} \frac{\partial G}{\partial w_{i}}\geq & 1-2\Delta te^{-2w_{i}}-\Delta tIf^{2}\left(\frac{f^{2}+x^{2}}{\sqrt{W+1}\;Q^{2}}\right)\;{\left|\hat{w}\right|}_{\max}\left|\frac{\partial {\hat{w}}_{x,i}}{\partial w_{i}}\right|\;\\ = & 1-2\Delta te^{-2w_{i}}-\Delta t\frac{I}{\sqrt{W+1}}{\left(f^{2}+x^{2}\right)}^{2}{\left|\hat{w}\right|}_{\max}\cdot \left|\frac{1}{\Delta x}\right| \end{array}$$

Hence, we can formulate a sufficient condition for monotonicity on the time step size as follows:

(67) $$\begin{array}{ccc} \frac{\partial G}{\partial w_{i}}\geq 0 & \Leftrightarrow & 1-2\Delta te^{-2w_{i}}-\Delta t\frac{I}{\sqrt{W+1}}{\left(f^{2}+x^{2}\right)}^{2}{\left|\hat{w}\right|}_{\max}\cdot \left|\frac{1}{\Delta x}\right|\geq 0\;\\ & \Leftrightarrow & 2\Delta te^{-2w_{i}}+\Delta t\frac{I}{\sqrt{W+1}}{\left(f^{2}+x^{2}\right)}^{2}{\left|\hat{w}\right|}_{\max}\cdot \frac{1}{\Delta x}\leq 1\;\\ & \Leftrightarrow & \Delta t\leq \frac{1}{2e^{-2w_{i}}+\frac{I{\left(f^{2}+x^{2}\right)}^{2}}{\sqrt{W+1}}\cdot \frac{{\left|\hat{w}\right|}_{\max}}{\Delta x}} \end{array}$$

As a consequence, for the Lambertian case, the scheme satisfies monotonicity and, therefore, stability as long as (67) is valid.

*Oren–Nayar case* ($H^{ON}=If^{2}F_{ON}\left(W\right)=\left(If^{2}\right)\frac{W+1}{A\sqrt{W+1}+BW}$)

(68) $$\begin{array}{ccc} \frac{\partial G}{\partial w_{i}} & = & 1-2\Delta t\;e^{-2w_{i}}-\Delta t\frac{\partial H^{ON}}{\partial w_{i}}\\ \; & = & 1-2\Delta t\;e^{-2w_{i}}\\ \; & \; & -\Delta tIf^{2}\left(\frac{\frac{\partial W}{\partial w_{i}}\left(A\sqrt{W+1}+BW\right)-\left(W+1\right)\left[\frac{A}{2\sqrt{W+1}}\frac{\partial W}{\partial w_{i}}+B\frac{\partial W}{\partial w_{i}}\right]}{{\left(A\sqrt{W+1}+BW\right)}^{2}}\right)\\ \; & = & 1-2\Delta t\;e^{-2w_{i}}-\Delta tIf^{2}\left(\frac{2(f^{2}+x^{2})}{Q^{2}}\right){\hat{w}}_{x,i}\\ \; & \; & \cdot \frac{\partial {\hat{w}}_{x,i}}{\partial w_{i}}\left(\frac{A\sqrt{W+1}+BW-\left(W+1\right)\left(\frac{A}{2\sqrt{W+1}}\frac{\partial W}{\partial w_{i}}+B\frac{\partial W}{\partial w_{i}}\right)}{{\left(A\sqrt{W+1}+BW\right)}^{2}}\right)\\ \; & = & 1-2\Delta t\;e^{-2w_{i}}-\Delta tI\;2{\left(f^{2}+x^{2}\right)}^{2}\;\;{\hat{w}}_{x,i}\frac{\partial {\hat{w}}_{x,i}}{\partial w_{i}}\underset{\geq 0\;\;\mathrm{if}\;\;A/2>B}{\underset{︸}{\left(\frac{\frac{A}{2}\sqrt{W+1}-B}{{\left(A\sqrt{W+1}+BW\right)}^{2}}\right)}} \end{array}$$

Recalling the definition ${\hat{w}}_{x,i}:=\max\left(0,-D^{+}w,D^{-}w\right)$ and the estimate (65) used in the previous Lambertian model case, we arrive at the following condition:

(69) $$\begin{array}{ccc} \; & \; & \frac{\partial G}{\partial w_{i}}\geq 0\\ \; & \Leftrightarrow & 1-2\Delta te^{-2w_{i}}-2\;\Delta tI{\left(f^{2}+x^{2}\right)}^{2}{\left|\hat{w}\right|}_{\max}\cdot \left|\frac{1}{\Delta x}\right|\left(\frac{\frac{A}{2}\sqrt{W+1}-B}{{\left(A\sqrt{W+1}+BW\right)}^{2}}\right)\geq 0\;\\ \; & \Leftrightarrow & \Delta t\leq \frac{1}{2}\frac{1}{e^{-2w_{i}}+I{\left(f^{2}+x^{2}\right)}^{2}\cdot \frac{{\left|\hat{w}\right|}_{\max}}{\Delta x}\;\left(\frac{\frac{A}{2}\sqrt{W+1}-B}{{\left(A\sqrt{W+1}+BW\right)}^{2}}\right)} \end{array}$$

Hence, for the Oren–Nayar case, the scheme satisfies monotonicity and, therefore, stability as long as (69) is valid.

*Blinn–Phong case* ($H^{BP}\;=\;\left(I-I_{a}k_{a}\right)f^{2}F_{BP}\left(W\right)\;=\;\left(I-I_{a}k_{a}\right)f^{2}\frac{{\left(W+1\right)}^{c/2}}{k_{D}{\left(W+1\right)}^{\frac{c-1}{2}}+k_{S}}$, with c≥1)

Let us define $DEN_{BP}:=\left(k_{D}{\left(W+1\right)}^{\frac{c-1}{2}}+k_{S}\right)$.

We start focusing on $\frac{\partial F_{BP}}{\partial w_{i}}$:

(70) $$\begin{array}{ccc} \; & \; & \frac{\partial F_{BP}}{\partial w_{i}}=\frac{\frac{c}{2}{\left(W+1\right)}^{\frac{c}{2}-1}\cdot \frac{\partial W}{\partial w_{i}}\cdot DEN_{BP}-k_{D}\frac{c-1}{2}{\left(W+1\right)}^{\frac{2c-3}{2}}\cdot \frac{\partial W}{\partial w_{i}}}{DEN_{BP}^{2}}\\ \; & = & \frac{\partial W}{\partial w_{i}}\left(\frac{\frac{c}{2}{\left(W+1\right)}^{\frac{c}{2}-1}k_{D}{\left(W+1\right)}^{\frac{c-1}{2}}+k_{S}\frac{c}{2}{\left(W+1\right)}^{\frac{c}{2}-1}-k_{D}\frac{c-1}{2}{\left(W+1\right)}^{\frac{2c-3}{2}}}{DEN_{BP}^{2}}\right)\\ \; & = & \left(\frac{2(f^{2}+x^{2})}{Q^{2}}\right){\hat{w}}_{x,i}\frac{\partial {\hat{w}}_{x,i}}{\partial w_{i}}\left(\frac{k_{S}\frac{c}{2}{\left(W+1\right)}^{\frac{c}{2}-1}+{\left(W+1\right)}^{\frac{2c-3}{2}}\left(k_{D}\frac{c}{2}-k_{D}\frac{c-1}{2}\right)}{DEN_{BP}^{2}}\right)\\ \; & = & \left(\frac{2{\left(f^{2}+x^{2}\right)}^{2}}{f^{2}}\right){\hat{w}}_{x,i}\frac{\partial {\hat{w}}_{x,i}}{\partial w_{i}}\underset{>0}{\underset{︸}{\left(\frac{k_{S}\frac{c}{2}{\left(W+1\right)}^{\frac{c}{2}-1}+\frac{k_{D}}{2}{\left(W+1\right)}^{\frac{2c-3}{2}}}{DEN_{BP}^{2}}\right)}} \end{array}$$

Since the last term is positive, recalling the estimate (65) where ${\hat{w}}_{x,i}:=\max$$\left(0,-D^{+}w,D^{-}w\right)$, we can finally write the following:

(71) $$\begin{array}{ccc} \; & \; & \frac{\partial G}{\partial w_{i}}=1-2\Delta t\;e^{-2w_{i}}-\Delta t\frac{\partial H^{BP}}{\partial w_{i}}\;\\ \; & = & 1-2\Delta t\;e^{-2w_{i}}-\Delta t\left(I-I_{a}k_{a}\right)f^{2}\frac{\partial F_{BP}}{\partial w_{i}}\;\\ \; & = & 1-2\Delta t\;e^{-2w_{i}}-\Delta t\left(I-I_{a}k_{a}\right)f^{2}\;\left(\frac{2{\left(f^{2}+x^{2}\right)}^{2}}{f^{2}}\right){\hat{w}}_{x,i}\;\\ \; & \; & \cdot \frac{\partial {\hat{w}}_{x,i}}{\partial w_{i}}\left(\frac{k_{S}\frac{c}{2}{\left(W+1\right)}^{\frac{c}{2}-1}+\frac{k_{D}}{2}{\left(W+1\right)}^{\frac{2c-3}{2}}}{DEN_{BP}^{2}}\right)\;\\ \; & \geq & 1-2\Delta t\;e^{-2w_{i}}-\Delta t\left(I-I_{a}k_{a}\right)\;\left(2{\left(f^{2}+x^{2}\right)}^{2}\right){\left|\hat{w}\right|}_{\max}\;\\ \; & \; & \cdot \left|\frac{1}{\Delta x}\right|\left(\frac{k_{S}\frac{c}{2}{\left(W+1\right)}^{\frac{c}{2}-1}+\frac{k_{D}}{2}{\left(W+1\right)}^{\frac{2c-3}{2}}}{DEN_{BP}^{2}}\right)\;\\ \; & = & 1-2\Delta t\left(e^{-2w_{i}}+\left(I-I_{a}k_{a}\right)\;{\left(f^{2}+x^{2}\right)}^{2}{\left|\hat{w}\right|}_{\max}\right.\;\\ \; & \; & \left.\cdot \left|\frac{1}{\Delta x}\right|\left(\frac{k_{S}\frac{c}{2}{\left(W+1\right)}^{\frac{c}{2}-1}+\frac{k_{D}}{2}{\left(W+1\right)}^{\frac{2c-3}{2}}}{DEN_{BP}^{2}}\right)\right) \end{array}$$

Hence,

(72) $$\;\begin{array}{ccc} \frac{\partial G}{\partial w_{i}} & \geq & 0\Leftrightarrow \\ \Delta t & \leq & \frac{1}{2}\frac{1}{\left(e^{-2w_{i}}+\left(I-I_{a}k_{a}\right)\;{\left(f^{2}+x^{2}\right)}^{2}{\left|\hat{w}\right|}_{\max}\cdot \left|\frac{1}{\Delta x}\right|\left(\frac{k_{S}\frac{c}{2}{\left(W+1\right)}^{\frac{c}{2}-1}+\frac{k_{D}}{2}{\left(W+1\right)}^{\frac{2c-3}{2}}}{DEN_{BP}^{2}}\right)\right)} \end{array}$$

which guarantees monotonicity and, therefore, stability for the Blinn–Phong case.

*Phong case* $\left(F_{PH}\left(W\right)=\left\{\begin{array}{cc} \frac{\sqrt{W+1}}{k_{D}} & \mathrm{if}\;W\geq 1\\ \frac{{\left(W+1\right)}^{\alpha +1/2}}{k_{D}{\left(W+1\right)}^{\alpha }+k_{S}{\left(W+1\right)}^{1/2}{\left(1-W\right)}^{\alpha }} & \mathrm{if}\;0\leq W<1 \end{array}\right.\right)$

In the case of W≥1, we fall back on the Lambertian case, which we have already seen. In the case 0≤W<1, let us start computing $\frac{\partial F_{PH}}{\partial w_{i}}$. We define $DEN:=k_{D}{\left(W+1\right)}^{\alpha }+k_{S}{\left(W+1\right)}^{1/2}{\left(1-W\right)}^{\alpha }$ for brevity.

(73) $$\begin{array}{ccc} \;\frac{\partial F_{PH}}{\partial w_{i}} & = & \frac{\left(\alpha +\frac{1}{2}\right){\left(W+1\right)}^{\alpha -\frac{1}{2}}\cdot \frac{\partial W}{\partial w_{i}}\cdot DEN-{\left(W+1\right)}^{\alpha +\frac{1}{2}}\cdot \frac{\partial DEN}{\partial w_{i}}}{DEN^{2}}\\ & = & \frac{1}{DEN^{2}}\left(\left(\alpha +\frac{1}{2}\right){\left(W+1\right)}^{\alpha -\frac{1}{2}}\cdot \frac{\partial W}{\partial w_{i}}\cdot DEN-{\left(W+1\right)}^{\alpha +\frac{1}{2}}\right.\\ & & \cdot \left.(\alpha k_{D}{\left(W+1\right)}^{\alpha -1}\frac{\partial W}{\partial w_{i}}+\frac{k_{S}}{2}{\left(W+1\right)}^{-1/2}\frac{\partial W}{\partial w_{i}}{\left(1-W\right)}^{\alpha }\right.\\ & & \left.+k_{S}{\left(W+1\right)}^{1/2}\alpha {\left(1-W\right)}^{\alpha -1}\frac{\partial W}{\partial w_{i}}\left(-1\right))\right)\\ & = & \frac{1}{DEN^{2}}\;\frac{\partial W}{\partial w_{i}}\left(\left(\alpha +\frac{1}{2}\right){\left(W+1\right)}^{\alpha -\frac{1}{2}}\cdot DEN-\alpha k_{D}{\left(W+1\right)}^{2\alpha -\frac{1}{2}}\right.\\ & & \left.-\frac{k_{S}}{2}{\left(W+1\right)}^{\alpha }{\left(1-W\right)}^{\alpha }+\alpha k_{S}{\left(W+1\right)}^{\alpha +1}{\left(1-W\right)}^{\alpha -1}\right)\\ & = & \frac{1}{DEN^{2}}\;\frac{\partial W}{\partial w_{i}}\left(\left(\alpha +\frac{1}{2}\right){\left(W+1\right)}^{\alpha -\frac{1}{2}}\cdot k_{D}{\left(W+1\right)}^{\alpha }\right.\\ & & \left.+\left(\alpha +\frac{1}{2}\right){\left(W+1\right)}^{\alpha -\frac{1}{2}}\;k_{S}{\left(W+1\right)}^{\frac{1}{2}}{\left(1-W\right)}^{\alpha }-\alpha k_{D}{\left(W+1\right)}^{2\alpha -\frac{1}{2}}\right.\\ & & -\left.\frac{k_{S}}{2}{\left(W+1\right)}^{\alpha }{\left(1-W\right)}^{\alpha }+\alpha k_{S}{\left(W+1\right)}^{\alpha +1}{\left(1-W\right)}^{\alpha -1}\right)\\ & = & \frac{1}{DEN^{2}}\;\frac{\partial W}{\partial w_{i}}\left({\left(W+1\right)}^{2\alpha -\frac{1}{2}}\left(\left(\alpha +\frac{1}{2}\right)k_{D}-\alpha k_{D}\right)\right.\\ & & \left.+{\left(W+1\right)}^{\alpha +1}{\left(1-W\right)}^{\alpha -1}\alpha k_{S}+{\left(W+1\right)}^{\alpha }{\left(1-W\right)}^{\alpha }\left(k_{S}\left(\alpha +\frac{1}{2}\right)-\frac{k_{S}}{2}\right)\right)\\ & = & \frac{1}{DEN^{2}}\;\frac{\partial W}{\partial w_{i}}\left(\left(\frac{k_{D}}{2}\right){\left(W+1\right)}^{2\alpha -\frac{1}{2}}\right.\\ & & \left.+\alpha k_{S}\left({\left(W+1\right)}^{\alpha +1}{\left(1-W\right)}^{\alpha -1}+{\left(W+1\right)}^{\alpha }{\left(1-W\right)}^{\alpha }\right)\right) \end{array}$$

Hence, we obtain

(74) $$\begin{array}{ccc} \;\frac{\partial G}{\partial w_{i}} & = & 1-2\Delta t\;e^{-2w_{i}}-\Delta t\frac{\partial H^{PH}}{\partial w_{i}}\\ \; & = & 1-2\Delta t\;e^{-2w_{i}}-\Delta t\left(I-I_{a}k_{a}\right)f^{2}\frac{\partial F_{PH}}{\partial w_{i}}\\ \; & = & 1-2\Delta t\;e^{-2w_{i}}-\Delta t\frac{\left(I-I_{a}k_{a}\right)f^{2}}{DEN^{2}}\;\frac{\partial W}{\partial w_{i}}\\ \; & \; & \;\cdot \left((\underset{\geq 0}{\underset{︸}{\frac{k_{D}}{2}}})\underset{>0}{\underset{︸}{{\left(W+1\right)}^{2\alpha -\frac{1}{2}}}}+\underset{\geq 0}{\underset{︸}{\alpha k_{S}}}\underset{>0}{\underset{︸}{\left({\left(W+1\right)}^{\alpha +1}{\left(1-W\right)}^{\alpha -1}+{\left(W+1\right)}^{\alpha }{\left(1-W\right)}^{\alpha }\right)}}\right) \end{array}$$

Since the last term is (⋯)≥0, recalling the definition of ${\hat{w}}_{x,i}$ and the estimate (65), we can obtain the following:

(75) $$\;\begin{array}{ccc} \; & \; & \frac{\partial G}{\partial w_{i}}\geq 0\\ \; & \Leftrightarrow & 1-2\Delta t\left(e^{-2w_{i}}+\frac{\left(I-I_{a}k_{a}\right)}{DEN^{2}}\;{\left(f^{2}+x^{2}\right)}^{2}{\left|\hat{w}\right|}_{\max}\cdot \left|\frac{1}{\Delta x}\right|\right.\\ \; & \; & \;\cdot \left.\left(\left(\frac{k_{D}}{2}\right){\left(W+1\right)}^{2\alpha -\frac{1}{2}}+\alpha k_{S}\left({\left(W+1\right)}^{\alpha +1}{\left(1-W\right)}^{\alpha -1}+{\left(W+1\right)}^{\alpha }{\left(1-W\right)}^{\alpha }\right)\right)\right)\geq 0\\ \; & \Leftrightarrow & \Delta t\leq \frac{1}{2}\frac{1}{\left(e^{-2w_{i}}\;+\;\frac{\left(I-I_{a}k_{a}\right)}{DEN^{2}}\;{\left(f^{2}+x^{2}\right)}^{2}{\left|\hat{w}\right|}_{\max}\;\cdot \;\left|\frac{1}{\Delta x}\right|\left(\left(\frac{k_{D}}{2}\right){\left(W+1\right)}^{2\alpha -\frac{1}{2}}\;+\;\alpha k_{S}T_{PH}\right)\right)} \end{array}$$

with $T_{PH}:=\left({\left(W+1\right)}^{\alpha +1}{\left(1-W\right)}^{\alpha -1}\;+\;{\left(W+1\right)}^{\alpha }{\left(1-W\right)}^{\alpha }\right)$. Hence, the monotonicity and, therefore, stability is also guaranteed for the Phong case. □

**Remark** **3.** *Note that the convergence result is mainly based on the signs of the common function W(x,p) and the different functions $F_{M}$, with M, indicating the acronym of the four models (M=L,ON,PH,BP), with their related properties. Hence, the convergence of the numerical scheme still holds in the case of possible imperfect knowledge of terms such as the percentage of the ambient component $k_{A}$.*

## 4. Numerical Simulations

In this section, we report some one-dimensional synthetic numerical experiments, which confirm the convergence of the proposed scheme to a viscosity solution. All the numerical tests were implemented in Matlab, by using a Notebook HP EliteBook 830 G6 Intel Core i5-8265U with a speed of 1.60 GHz and 16 GB of RAM.

**Test 1.**

The unknown function *u* we want to reconstruct is defined by the following equation:

(76) $$u\left(x\right)=x^{2}+3\;x\in \left[-0.5,0.5\right].$$

**Figure 1:**
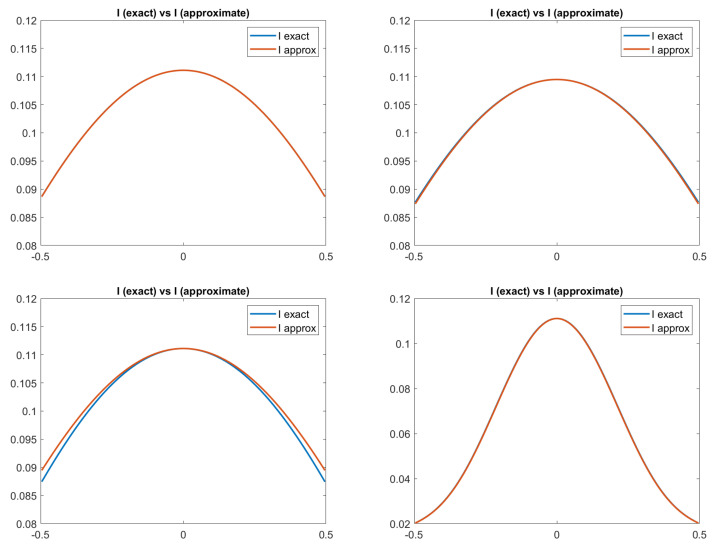
Test 1. Plots of the exact and approximated images related to the four reflectance models. From top-left to bottom-right: images related to Lambertian model; ON-model with σ = 0.1; PH-model with $k_{A}\;=\;0,\;k_{D}\;=\;0.8,\;k_{S}\;=\;0.2,\;\alpha \;=\;2$; BP-model with $k_{A}\;=\;0,\;k_{D}\;=\;0.2,\;k_{S}\;=\;0.8,\;c\;=\;50$.

Since it is a synthetic case, the input image is different for each model, generated by the proper brightness equation. This is clearly visible in Figure 1, where the true and the approximated images for each model are reported. The approximated four image functions *I* are computed a posteriori using the *u* computed by the scheme and central finite difference to obtain the gradient of *u*.

**Figure 2:**
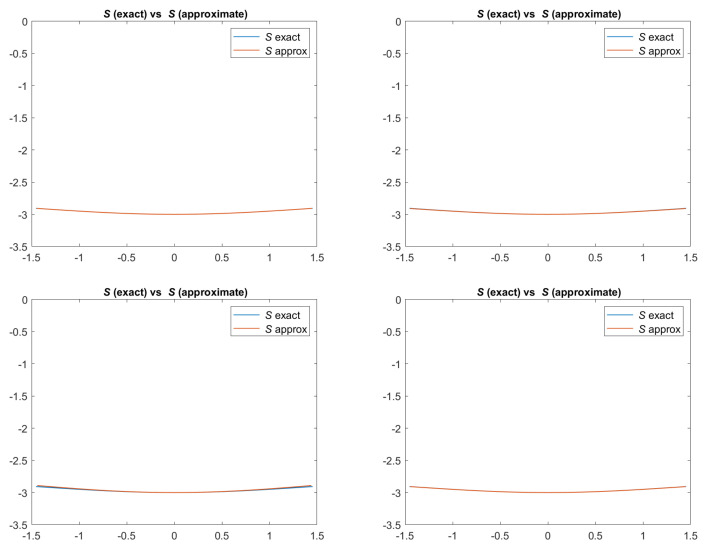
Test 1. Plots of the exact and approximated scenes defined according to Equation (3) and related to the four reflectance models. From top-left to bottom-right: images related to Lambertian model; ON-model with σ = 0.1; PH-model with $k_{A}\;=\;0,\;k_{D}\;=\;0.8,\;k_{S}\;=\;0.2,\;\alpha \;=\;2$; BP-model with $k_{A}\;=\;0,k_{D}\;=\;0.2,\;k_{S}\;=\;0.8,\;c\;=\;50$.

In Figure 2, we can see the exact and the approximated scenes defined according to Equation (3). For all the models, the approximation is really good.

**Figure 3:**
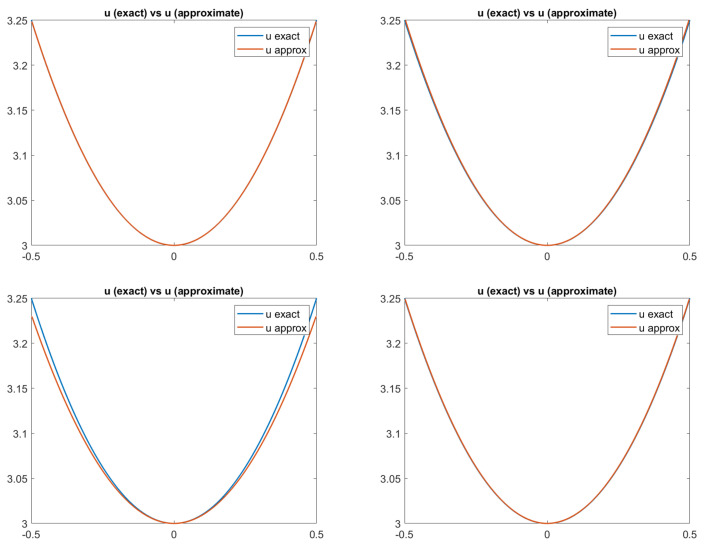
Test 1. Plots of the exact and approximated heights of the surfaces related to the four reflectance models. From top-left to bottom-right: images related to Lambertian model; ON-model with σ = 0.1; PH-model with $k_{A}\;=\;0,\;k_{D}\;=\;0.8,\;k_{S}\;=\;0.2,\;\alpha =2$; BP-model with $k_{A}\;=\;0,k_{D}\;=\;0.2,$ $k_{S}\;=\;0.8,\;c\;=\;50$.

**Figure 4:**
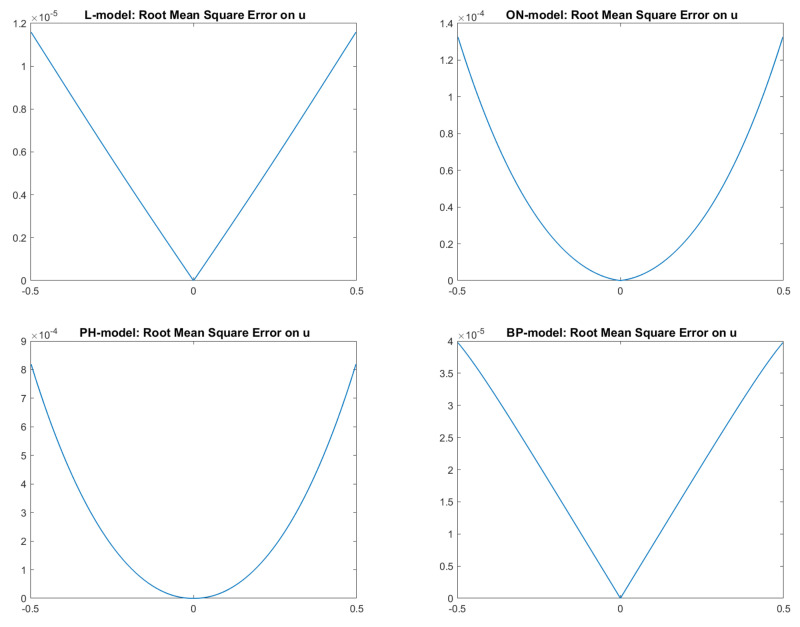
Test 1. Plots of the RMSEs in the four cases. From top-left to bottom-right: images related to Lambertian model; ON-model with σ=0.1; PH-model with $k_{A}\;=\;0,\;k_{D}\;=\;0.8,\;k_{S}\;=\;0.2,\;\alpha \;=\;2$; BP-model with $k_{A}\;=\;0,\;k_{D}\;=\;0.2,\;k_{S}\;=\;0.8,\;c\;=\;50$.

Finally, in Figure 3, we report the plots of the exact and approximated heights of the surfaces related to each of the four reflectance models. Qualitatively, in all four cases, the solution computed by the scheme seems to be a good approximation of the surface height, confirming that the numerical solution converges to the correct (and unique) viscosity solution. In fact, looking at the pictures reported in Figure 3, it is difficult to distinguish the exact solution from the approximate one, except for in the PH-model, where it differs going towards the boundary. In order to also perform a quantitative analysis of these results, we compute the Root Mean Square Errors (RMSEs) related to the solutions of the four cases for each point, as shown in Figure 4 and defined as follows:

(77) $$Error\left(i\right)=\sqrt{\frac{1}{N}\;{\left(v\left(x_{i}\right)-w_{i}\right)}^{2}},\;\mathrm{for}\;\;\;i=1,\cdots ,N.$$

Analyzing those figures, we can state that the RMSEs are of order $10^{-5}$ for the L-model and BP-model, whereas they are are of order $10^{-4}$ for the ON-model and PH-model.

**Test 2.**

For this second synthetic test, let us consider the following function:

(78) u(x)=cos(5x)+5x∈[−0.8,0.1].

**Figure 5:**
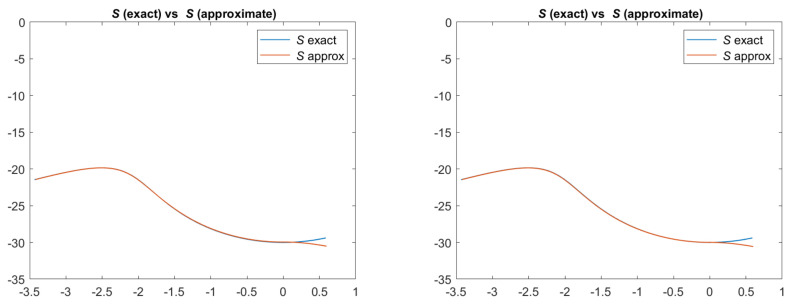
Test 2. Plots of the exact and approximated scenes related to two reflectance models. From left to right: scenes related to PH-model and BP-model, with the parameters $k_{a}\;=\;0,\;k_{D}\;=\;0.8,\;k_{S}\;=\;0.2,$ α = 10, c = 50. Focal length f = 5.

**Figure 6:**
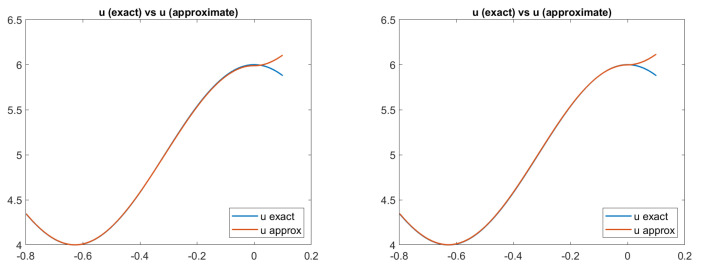
Test 2. Plots of the exact and approximated heights of the surfaces related to two reflectance models. From left to right: surface heights related to PH-model and BP-model, with the parameters $k_{a}\;=\;0,\;k_{D}\;=\;0.8,\;k_{S}\;=\;0.2,$ α = 10, c = 50. Focal length f = 5.

**Figure 7:**
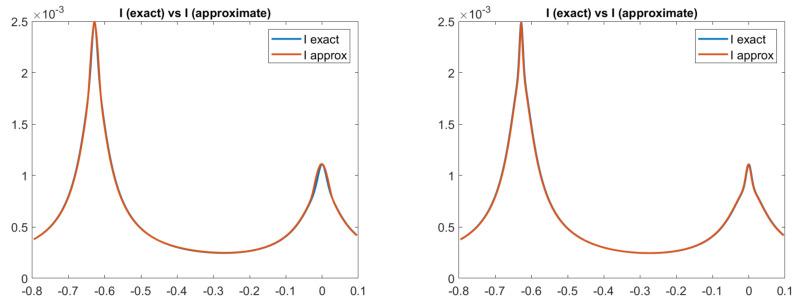
Test 2. Plots of the exact and approximated images related to two reflectance models. From left to right: images related to PH-model and BP-model, with the parameters $k_{a}\;=\;0,\;k_{D}\;=\;0.8,\;k_{S}\;=\;0.2,$ α = 10, c = 50. Focal length f = 5.

**Figure 8:**
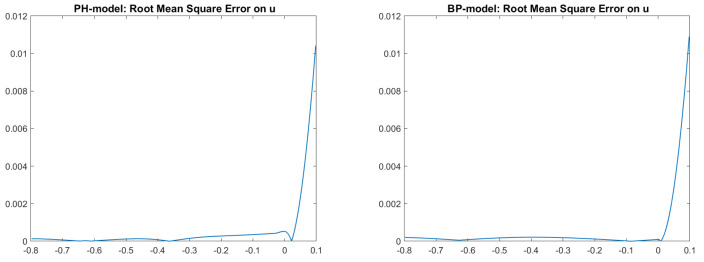
Test 2. Plots of the RMSEs related to the two cases reported in Figure 6. From left to right: images related to PH-model and BP-model, with the parameters $k_{a}\;=\;0,k_{D}\;=\;0.8,\;k_{S}\;=\;0.2,$ α = 10, c = 50. Focal length f = 5.

Looking at Figure 5 and Figure 6, we can see that an ambiguity, which remembers a concave/convex ambiguity typical of an orthographic setup, is present on the right side of the scene, even if the image is perfectly reconstituted, as shown, for example, in Figure 7 for the Phong and Blinn–Phong models. Looking at Figure 8, we can note that for both models the error is close to zero, except on the right side of the pictures, in correspondence to the ambiguity, where there is an error of about 0.01.

The ambiguity of this particular case is due to the model, not to the specific numerical scheme chosen to solve the problem. The existence of different surfaces which share the same image does not contradict the uniqueness result related to the viscosity solution, since they can correspond to different boundary conditions, or to the same boundary conditions imposed in a different domain of definition. This is just to stress that the uniqueness of the viscosity solution does not completely solve the problem of model ambiguity, since we could be interested in reconstructing a surface described by a weak solution, which is different from the viscosity solution. We refer the reader to [36] for more details on the ambiguities of the model. The mentioned paper refers only to the Lambertian case, but the analysis performed, even if only on a single model, is enough to understand what happens.

## 5. Conclusions

In this paper, we have shown a convergence result for the recent unified formulation of the perspective SfS models proposed in [29], in which the authors considered an attenuation term in order to achieve the well-posedness of the problem in the context of viscosity solutions. Despite the peculiarities of the different reflectance models considered, by formulating them in a unified framework described by a general Hamiltonian, which has attractive properties, we proved that a monotone, consistent and stable explicit scheme of upwind type converges to a viscosity solution, under uniqueness properties of the Hamiltonian. This result is interesting and powerful since can be easily extended to other non-Lambertian models in a unified framework. The numerical experiments shown confirm the convergence of the proposed scheme. We believe that this work could help to develop convergent numerical methods of refined SfS processes for real-world scenarios in future research.
